# Malaria morbidity and mortality trends in Manicaland province, Zimbabwe, 2005-2014

**DOI:** 10.11604/pamj.2017.27.30.11130

**Published:** 2017-05-11

**Authors:** Faith Mutsigiri, Patron Trish Mafaune, More Mungati, Gerald Shambira, Donewell Bangure, Tsitsi Juru, Notion Tafara Gombe, Mufuta Tshimanga

**Affiliations:** 1 Department of Community Medicine, University of Zimbabwe, Harare, Zimbabwe; 2 Ministry of Health and Child Care, Manicaland Province, Zimbabwe

**Keywords:** Malaria morbidity and mortality, trends, Manicaland, Zimbabwe

## Abstract

**Introduction:**

Zimbabwe targets reducing malaria incidence from 22/1000 in 2012 to 10/1000 by 2017, and malaria deaths to near zero by 2017. As the country moves forward with the malaria elimination efforts, it is crucial to monitor trends in malaria morbidity and mortality in the affected areas. In 2013, Manicaland Province contributed 51% of all malaria cases and 35% of all malaria deaths in Zimbabwe. This analysis describes the trends in malaria incidence, case fatality and malaria outpatient workload compared to the general outpatient workload.

**Methods:**

We analyzed routinely captured malaria data in Manicaland Province for the period 2005 to 2014. Epi Info version 7 was used to calculate chi-square trends for significance and Microsoft Excel was used to generate graphs. Permission to analyze the data was sought and granted by the Provincial Medical Directorate Institutional Review Board of Manicaland and the Health Studies office.

**Results:**

Malaria morbidity data for the period 2005-2014 was reviewed and a total of 947,462 cases were confirmed during this period. However, malaria mortality data was only available for the period 2011-2014 and cumulatively 696 deaths were reported. Malaria incidence increased from 4.4/1,000 persons in 2005 to 116.3/1,000 persons in 2014 (p<0.001). The incidence was higher among females compared to males (p-trend<0.001) and among the above five years age group compared to the under-fives (p-trend<0.001). The proportion of all Outpatient Department attendances that were malaria cases increased 30 fold from 0.3% in 2005 to 9.1% in 2014 (p-trend<0.001). The Case Fatality Rate also increased 2-fold from 0.05 in 2011 to 0.1 in 2014 (p-trend<0.001).

**Conclusion:**

Despite current malaria control strategies, the morbidity and mortality of malaria increased over the period under review. There is need for further strengthening of malaria control interventions to reduce the burden of the disease.

## Introduction

Malaria is among the world's commonest and life threatening tropical diseases. It is commonly caused by *Plasmodium falciparum* parasites which are transmitted through a bite from an infected female Anopheles mosquito which occurs mainly between dusk and dawn [1]. Malaria is endemic in most tropical regions and of the estimated 3.4 billion people worldwide who are exposed to malaria annually, 1.2 billion are at high risk [1]. Although preventable and curable, malaria causes significant morbidity and mortality especially in regions with limited resources [2]. In 2014, 97 countries and territories had on going malaria transmission throughout the year [1]. An estimated 300-500 million people contract clinical malaria each year and 1.5-2.7 million deaths occur annually [3, 4]. Of all these malaria deaths, about one million are children below 5 years of age [5]. Sub-Saharan Africa is the most affected region [2] contributing over 80% of global malaria deaths [6]. In sub-Saharan Africa, malaria is responsible for 30-50% of all outpatient visits in clinics and up to 50% of hospital admissions [7]. Malaria remains one of the top three causes of child mortality in Zimbabwe with approximately 1 in 12 children in Zimbabwe dying before their 5th birthday [8]. *Plasmodium falciparum* accounts for 98% of cases seen within health facilities in Zimbabwe. Plasmodium ovale and *Plasmodium malariae* account for the remaining 2% [9]. The malaria incidence peaks between February and May due to higher temperatures during this period. These high temperatures facilitate mosquito breeding. Rainfall directly translates to malaria endemicity, therefore Southern and Western parts of the country which receive little rainfall have lower prevalence of malaria [10]. Malaria is mainly seasonal in Zimbabwe, with potential epidemics during the rainy season [11]. Population immunity is affected by different transmission rates, thus, in high transmission zones fewer symptoms appear, and children with immature immune systems are threatened by the presence of asymptomatic carriers. People have low immunity and malaria is epidemic in areas where malaria control has been attempted [10]. The main goal of the National Malaria Control Programme is to reduce the current malaria incidence from 22/1000 in 2012 to 10/1000 by 2017 and malaria deaths to near zero by 2017 [9].

**Study setting**: The rainy season runs from November to April. The average minimum and maximum temperatures in Manicaland Province are 14 oC and 26 oC respectively. The area has many rivers and land use consists of farmlands, plantations for coffee/tea and banana. The province is administratively divided into seven districts with an estimated population of 1 752 698 according to the 2012 national census [12]. There are 283 health facilities and all routinely report malaria data. Malaria transmission in this region is seasonal and unstable, characterized by frequent epidemics with peaks from February to May.

**The malaria dataset**: In Zimbabwe malaria is a notifiable disease. The malaria dataset was set up for capturing the data on several malaria indicators to enable monitoring and evaluation, decision making and planning in the malaria control programme. Malaria case reporting is carried out using the Health Information Management System. Malaria data is reported weekly, monthly and quarterly. The data is collected daily by the health workers during management of outpatient attendees at the health facilities through the use of paper based tally sheets. The Village Health Workers also record this data during community case management. The data is then summarised on the paper based T5 monthly return form for onward transmission to the Health Information Officer at district level. The District Health Information Officer enters the data into the electronic District Health Information Software 2 (DHIS 2) which is internet based. At this point the data becomes available at all levels up to National level for analysis and consumption. The T5 forms remain available at District level thus data storage is both the paper based T5 monthly return forms and the electronic DHIS 2. Malaria indicators captured in the DHIS 2 include, the total number of suspected cases, total suspected cases tested using Rapid Diagnostic Tests (RDT) or blood slides and total number of confirmed cases (positive test result), which translates to malaria positivity rate. Malaria deaths are also captured in the DHIS 2 by health facility on a weekly basis. Since 2005, the malaria data is now being collected by sex and age.

## Methods

We analyzed retrospectively collected malaria data from Manicaland Province for the period 2005 to 2014. Malaria data captured in different databases was retrieved and used in the analysis. DHIS 2 started capturing data in 2011. Data for the period 2005 to 2010 was retrieved from the old and new T5 as well as DHIS 1.4 databases. Data was collected at the Provincial Health Information Department. For information that was not available at the provincial office, the District Health Information Departments were contacted via telephone for clarification and to get missing data. We used annual clinical malaria data reported for the seven districts covering the whole of Manicaland Province between 2005 and 2014. District population projections were used to calculate malaria incidence rates per 1,000 populations. Data were analyzed using Microsoft Office Excel 2007 to come up with graphs to demonstrate the various trends in malaria morbidity and mortality over the last 10 years. Chi - square test for trends and p-values for significance testing at 95% confidence level were generated using Epi info statistical package (version 7). Permission to analyse the data was sought and granted by the Provincial Medical Directorate Institutional Review Board of Manicaland and the Health Studies office. The study used de-identified data which was only used for the purposes of this study. Stored data lacked patient names thus no personal information was accessed in this study.

## Results

**Completeness of data**: Malaria morbidity data for the period 2005-2014 was reviewed. However malaria mortality data was reported beginning 2011. Data completeness of the T5 forms was assessed. During the period 2005-2014, the provincial average T5 completeness varied from 54% to 100%. The least values for all districts and the province at large were recorded in 2010.

**Figure 1:**
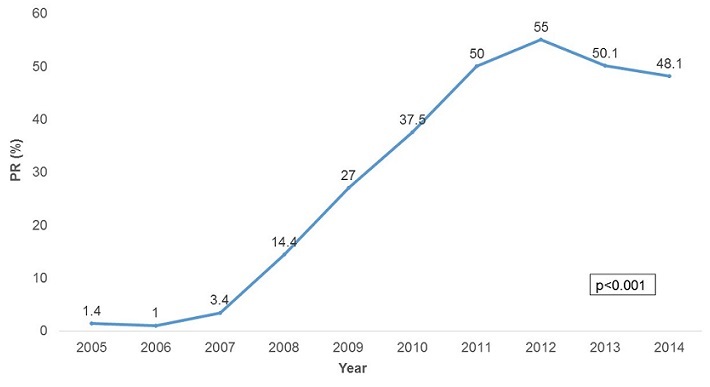
Malaria positivity rate trends by year in Manicaland province, Zimbabwe, 2005-2014

**Malaria Morbidity**: A total of 947,462 malaria cases were confirmed between 2005 and 2014. The majority of these cases were female (52.5%). In the last decade malaria was reported in all age groups, but the 5 years and above age group was the most affected constituting 82.0% of all cases. The malaria positivity rate increased from 1.4% in 2005 to 48.1% in 2014 with a peak of 55% in 2012 in the province. The increase was statistically significant (p-trend<0.001) Figure 1. There was an upward trend in malaria incidence in Manicaland Province in the last decade. The incidence was 4.4 cases per 1,000 persons in 2005 increasing to 116.3 cases per 1,000 persons in 2014 Figure 2. The incidence increased 26 fold during this period and the test for trend was highly significant (p-trend<0.001). The malaria incidences in all districts also showed an upward trend from 2005 to 2014. Buhera District had the lowest changes in malaria incidence. The incidence was 1.7 cases per 1,000 persons in 2005 increasing to 22.0 cases per 1,000 persons in 2014. The increase was statistically significant (Chi-square = 8 902.7, p<0.001). Nyanga District had the highest changes in incidence, increasing from 17.8 cases per 1,000 persons in 2005 to 288.9 cases per 1,000 persons in 2014. The test for trend was statistically significant (Chi-square = 80 505.0, p<0.001) The incidence of malaria among under-fives and those above five years increased from 8.4 to 113.2 cases per 1,000 persons and 3.6 to 124 cases per 1,000 respectively. The incidence of malaria was higher in under-fives from 2005 to 2007. From 2008 to 2014 the incidence was higher in those older than five years. The increase in incidence was significant for both the under-five and above five age groups (p-trend<0.001 for both) Figure 3. The incidence significantly increased from 4.4 to 120.4 cases per 1,000 persons between 2005 and 2014 among females (p<0.001). A similarly trend was noticed among males, increasing from 4.4 to 119.3 cases per 1,000 persons. This was also statistically significant (p<0.001). The case load within outpatients departments represented by malaria increased 30-fold from 0.3% in 2005 to 9.1% in 2014 with a peak of 15.7% in 2013. This increase was statistically significant (p<0.001) Figure 4.

**Figure 2:**
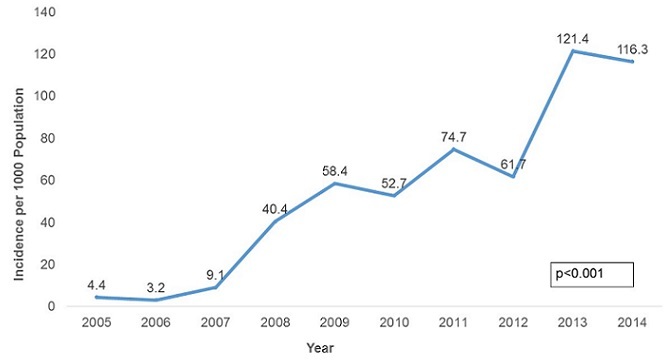
Malaria incidence trends in Manicaland province, Zimbabwe, 2005-2014

**Figure 3:**
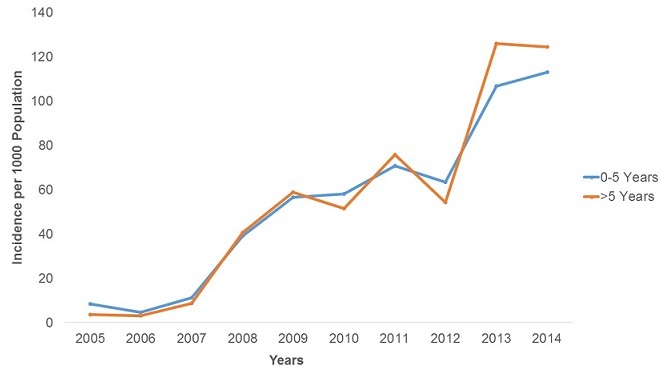
Malaria incidence by age in Manicaland province, Zimbabwe, 2005-2014

**Figure 4:**
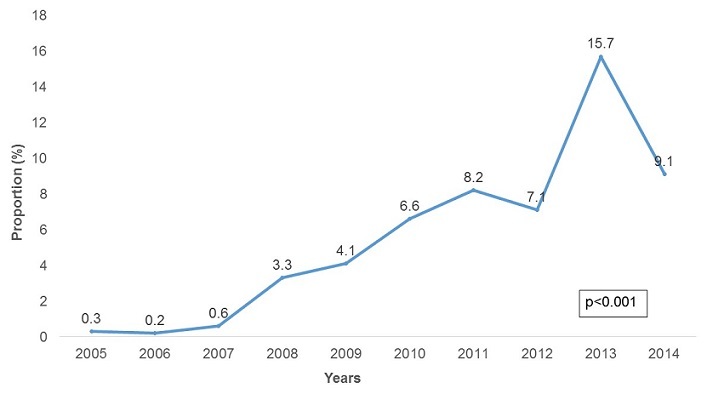
Proportion of malaria cases against all OPD cases by year, Manicaland province, Zimbabwe, 2005-2014

**Figure 5:**
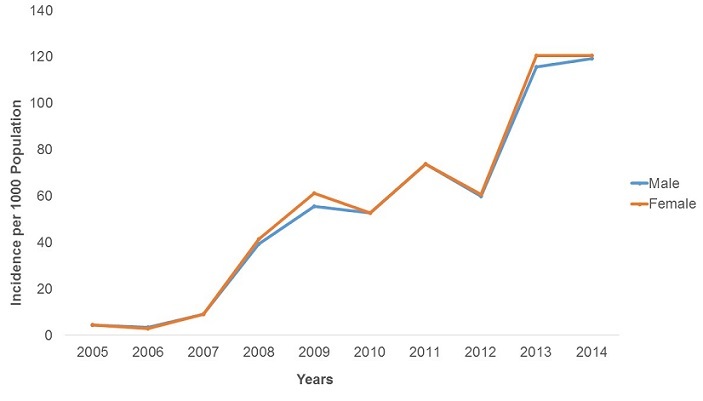
Malaria case fatality rate trends in Manicaland province, Zimbabwe, 2011-2014

**Figure 6:**
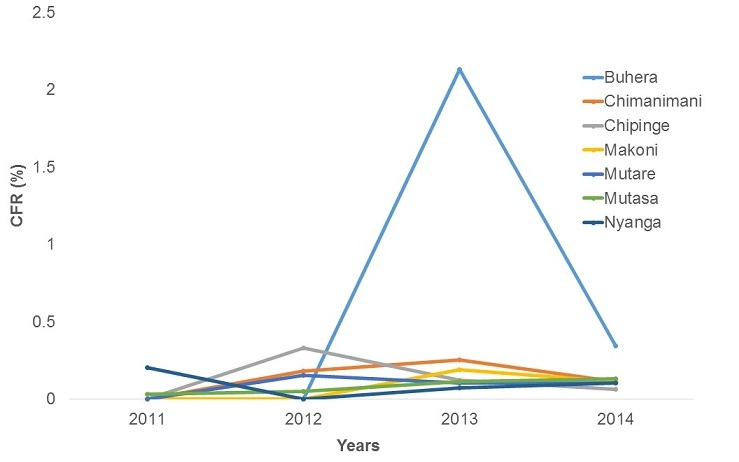
Malaria case fatality rate trends by district, Manicaland province, Zimbabwe, 2011 -2014

**Malaria Mortality**: The Cases Fatality Rate (CFR) in Manicaland Province increased from 0.05% in 2011 to 0.1% in 2014, with a peak in 2013 (CFR=0.14%). The increase was statistically significant (Chi-square = 15.1, p=0.0001) Figure 5. Chipinge District had the lowest changes in CFR. The CFR increased from 0% to 0.06% between 2011 and 2014. The increase was statistically significant (Chi-square = 47.7, p<0.001) Buhera District had the highest changes in incidence, increasing from 0% in 2005 to 0.34% in 2014. The test for trend was statistically significant (Chi-square = 10.6, p<0.001) Figure 6.

## Discussion

The analysis of malaria morbidity and mortality trends using surveillance data shows an overall increasing trend in confirmed malaria cases and deaths in the last decade in Manicaland Province. There was a significant rise in the number of confirmed cases noted from 2008. This can be attributed to the fact that RDT for malaria parasites were accessible in all health facilities from 2008 [13]. Policy states that all suspected malaria cases should be confirmed through parasitology. Initially focus was on malaria microscopy mainly done at the hospital level. Rolling out of RDTs has allowed extension of parasitological confirmation from the initial hospital (10%) primarily to cover all health facilities (approximately 100%). RDTs are easier to use and provide results faster hence testing rates are likely to increase and more accurate results yielded. Improved health worker ability to detect malaria cases using clinical symptoms due to training could have also contributed to the increase in RDT positivity rate. Those suspected of malaria were likely to test positive. Increasing malaria cases from 2007 may be attributed to resistance of malaria parasites to chloroquine. This is around the same time Coartem replaced chloroquine for the treatment of P. falciparum malaria together with Chloroquine and sulfadoxine-pyrimethamine. However, cases continued to increase until 2010 when ACT started being used as the first line treatment for malaria [13], subsequently there was a decrease in the number of malaria cases. Implementation of widespread use of artemisinin-based combination therapy (ACT) in the treatment of malaria has been shown to directly reduce malaria transmission, in addition to improving the malaria cure rates [14]. The increase in the number of malaria cases after introduction of more effective treatment (ACTs) noted from 2011 onwards may be assumed to be due to emerging resistance of P. falciparum parasites to ACT and/or pyrethroids used for Indoor residual Spraying (IRS).

Limitations in health facility based data in assessing trends in disease occurrence should be kept in mind especially service utilization and reporting completeness [15]. A decrease in the number of cases noted in 2010 may be partly attributed to the low completeness of surveillance data. The average provincial completeness in 2010 was 54%. Reduction in malaria incidence in 2010 also coincides with the optimal availability of the new effective medicine Coartem for the treatment of falciparum malaria. The reduction in malaria incidence may also be attributed to the increased awareness to malaria in communities resulting in increased use of preventive measures like Insecticide Treated Nets (ITNs). It can also be attributed to increased attention to malaria control and preventive strategies by organizations partnering with the Ministry of Health. Incidence rate varied widely between districts. Buhera and Makoni districts had the lowest incidence rates of malaria whilst Mutasa and Nyanga had the highest. This can be attributed to the climatic differences between these districts. Buhera and Makoni Districts are drier compared to Mutasa and Nyanga Districts. It has been shown that excessive rains increase malaria transmission by providing more mosquitoes breeding sites, leading to an increased malaria vector population [16, 17]. Buhera and Makoni districts have a flat terrain whereas Mutasa and Nyanga districts have bad terrains which make some communities inaccessible. Bad terrains may result in poor coverages of the IRS as spray teams fail to access some areas. As a result, the proportion of population protected and household coverage is reduced. Increasing the number of spray teams and spraying days may improve IRS coverages. The malaria incidence was higher in females compared to males. This finding might be due to different exposure rates. Females may be at risk due to work related activities in the fields where they are more likely to be exposed to mosquito bites than men. This finding is inconsistent with findings in other studies were a higher incidence rate was observed in males compared with females [18, 19]. It is necessary to point out that the relative proportion of females and males attending clinics in this study are comparable to the national census figure, indicating the absence of differential use of health care services across groups.

The 5 years and above age group had a higher incidence compared to under-fives. Adults and older children are at risk due to greater mobility and work related activities in the fields and risky areas. Younger children tend to stay indoors and travel less. They are also more likely to sleep under insecticide treated nets with their mothers. This is the same age group, together with pregnant women, which benefit from the ITN distribution program more than any other group. Similarly, in a study by Gerritsen et al (2008), incidence was lower in under-fives possibly because children at this age are usually indoors at night and benefit from the protection offered by IRS. As they get older, their sleeping habits may change and they may therefore be more at risk of infective mosquito bites [19]. Older ages have become more susceptible than before due to the gradual loss of partial immunity because of intensified malaria control. People are vulnerable in areas where malaria control has been attempted [10]. Zimbabwe is targeting malaria deaths to near zero by 2017 [9]. However, in this study malaria case fatality rate showed an increasing trend from 2011 to 2014. This can be attributed to increasing cases developing severe malaria which is a fatal and life threatening disease. Severe malaria can develop due to delayed care seeking by patients and poor case management by health workers. Findings of this study show that Buhera district has the highest CFR in the province despite it having the least incidence. The high CFR may reflect poor health care seeking behaviour, of people in the community, thinly distributed health facilities or logistical problems especially at peripheral clinics [19] Malaria is a huge public health problem due to the high morbidity and burden on health care facilities. Similarly in a study by Alemu et al (2012), malaria accounted for an increasing percentage of outpatient consultations in most health facilities in different regions of Ethiopia [20].

**Study Limitations**: Manicaland Province is habitat to many people of the apostolic sect who constitutes more than a third of the population. The members do not seek medical care at health facilities. It is therefore possible that some malaria cases and deaths among this group could have been missed by the passive reporting of malaria surveillance data, thereby underestimating the true malaria incidence and CFR.

## Conclusion

Despite current malaria control strategies, the morbidity and mortality of malaria increased over the period under review. Malaria incidence was higher in 2013 and 2014 than in other periods of the observation. Malaria incidence varies according to gender and age with females and those aged 5 years and above showing a significantly higher incidence. Mutasa and Nyanga Districts had higher incidence of malaria.

### What is known about this topic

- Malaria control strategies result in a decline in malaria morbidity and mortality.

### What this study adds

- Despite current malaria control strategies, the morbidity and mortality of malaria can still increase especially in resource limited areas like Zimbabwe.

## Competing interests

The author declare no competing interests.
